# Increasing Large Language Model Accuracy for Care-Seeking Advice Using Prompts Reflecting Human Reasoning Strategies in the Real World: Validation Study

**DOI:** 10.2196/88053

**Published:** 2026-04-08

**Authors:** Marvin Kopka, Markus A Feufel

**Affiliations:** 1 Division of Ergonomics, Department of Psychology & Ergonomics (IPA), Technische Universität Berlin, Straße des 17. Juni 135, Berlin, State of Berlin, 10623, Germany, 49 31470806

**Keywords:** prompting, human-technology interaction, human factors, artificial intelligence, decision-making, naturalistic decision-making, naturalistic decision support, cognitive science, care-seeking, self-triage, bounded rationality

## Abstract

**Background:**

Current prompting techniques for large language models (LLMs), such as ChatGPT, mainly focus on well-structured, low-uncertainty problems; yet, many real-world tasks (eg, care-seeking decisions) are ill-defined and involve high uncertainty. Naturalistic decision-making (NDM) specifically analyzes how humans make accurate decisions in such settings, but NDM concepts have not yet been applied to LLM prompt engineering.

**Objective:**

This study aimed to determine whether prompting strategies inspired by NDM (specifically based on recognition-primed decision-making and the data-frame theory) could improve LLM performance in a real-world, high-uncertainty task, such as making care-seeking decisions.

**Methods:**

We evaluated 10 ChatGPT models (GPT-4o, GPT-4.1, GPT-4.1 mini, o3, o4 mini, o4 mini high, GPT-5.1 Instant, GPT-5.1 Thinking, GPT-5.2 Instant, and GPT-5.2 Thinking) using 3 prompting strategies: a default prompt solely asking the LLMs to classify the case vignettes, a recognition-primed prompt tasking the models to reason according to recognition-primed decision-making, and a data-frame prompt tasking the models to apply the data-frame theory. The task was taken from a standardized and validated evaluation framework and instructed the LLMs to advise on the appropriate care-seeking action for 45 real patient case vignettes across 3 urgency levels (emergency, nonemergency, and self-care). Each model-vignette-prompt combination was tested 10 times to assess and account for output variability. Accuracy was analyzed using mixed effects logistic regression. Additionally, we evaluated accuracy for each urgency level and examined output variability.

**Results:**

Both NDM-inspired prompts increased overall model accuracy (recognition-primed: 67.6%; data-frame: 66.7%) compared to the default prompt (63.3%). The greatest improvements were observed for self-care recommendations, where accuracy increased from 13.4% (default prompt) to 29.8% (recognition-primed prompt) and 24.6% (data-frame prompt). Performance on 2 emergency and 30 nonemergency cases remained high across all prompts. Notably, NDM-inspired prompts made nonreasoning models start giving self-care advice, even though they rarely or never provided self-care advice with the default prompt. Output variability was similar across the 3 prompts.

**Conclusions:**

Using LLMs with prompts inspired by NDM, which are designed to reflect real-world human reasoning, improves the accuracy of LLMs in care-seeking tasks, particularly for self-care advice, without reducing performance in the included emergency or nonemergency cases. These findings indicate that NDM-inspired prompts can offer an advantage when LLMs are used for real-world decisions involving ambiguity and uncertainty. The impact of output that reflects real-world human reasoning on users’ decision-making must be evaluated in future studies.

## Introduction

Since their public release in 2022, large language models (LLMs), such as ChatGPT, have become widely used across domains for a range of tasks [1-8]. Although these models now reach high levels of accuracy on several benchmark tests, both researchers and users are increasingly interested in techniques to further improve model performance through specific input instructions—a process known as “prompting” [9]. Common approaches include assigning the model a specific role, providing relevant context or examples, or specifying a clear output format [10-12]. Three basic prompting strategies are often described in the literature: zero-shot, one-shot, and few-shot prompting. Zero-shot prompting refers to providing only the task instructions without any example outputs. One-shot prompting includes a single example of the expected output, and few-shot prompting provides multiple examples of the expected output [12]. In recent work, prompting strategies focus more on guiding the model through a reasoning process rather than simply providing information. A recent systematic review identified 58 prompting techniques, which were grouped into 6 categories [13]. In addition to zero-shot and few-shot approaches, 4 new categories were described: *ensembling prompts* use multiple prompts and aggregate the resulting outputs [13-16]. *Self-criticism prompts* instruct the model to evaluate and critique its own answers before responding [13,17,18]. *Decomposition prompts* instruct LLMs to break down tasks into smaller steps, which are then solved sequentially [13,19,20]. Finally, *thought generation* or “*chain-of-thought”* prompts ask the model to explicitly explain its reasoning as it works through a problem [13,21,22]. Notably, chain-of-thought and reasoning prompts have now been directly integrated into newer models [23]. For example, OpenAI’s o-series models (including o1, o3, and o4) are designed to generate a reasoning response before generating a user response [23]. This approach has been shown to improve accuracy across several benchmarks [21,24,25]. Starting with GPT-5, OpenAI also introduced a new, automatically included reasoning engine that consists of several internal expert models to which a user’s request is routed [26,27]. The model also automatically determines the reasoning effort needed to answer the user’s request [26,27].

Although the chain-of-thought prompting strategy is inspired by human reasoning, particularly deductive decision-making, there is an ongoing debate about whether LLMs really replicate human reasoning or simply generate plausible-sounding explanations [28]. Shojaee et al [29] recently tested chain-of-thought reasoning models on increasingly complex puzzles and found that LLMs do not engage in consistent reasoning across similar problems. In response, Lawsen [30] argues that these findings can be attributed to experimental artifacts and that LLMs are indeed capable of reasoning consistently and accurately when experimental setups are properly designed. Regardless of whether LLMs truly mimic human deductive reasoning when prompted with reasoning techniques, using human decision-making as a source of inspiration for developing prompting strategies is a promising direction. This is especially true in situations with high uncertainty, where deductive reasoning quickly reaches its limits, but humans are nonetheless able to make fairly good decisions [31].

Insights from the fields of applied psychology and human factors and ergonomics (HF/E) suggest that there is a gap between how humans reason in real-world situations and the assumed standard reasoning approaches related to deduction and induction, which are typically used to instruct and evaluate LLMs [28]. One explanation for this difference may be that humans often make decisions under uncertainty, with incomplete or ambiguous information, and decision tasks and goals are often ill-structured [32-34]. In contrast, most psychology experiments, as well as the current benchmarks for LLMs, rely on well-structured, multiple-choice tasks where all necessary information is explicitly provided, and the LLMs are merely asked to choose the correct answer out of multiple options, which can be readily evaluated against a clear-cut gold standard solution [35-39]. This test format is also used in most educational assessments, and LLMs perform well on this format—for example, passing professional and board certification exams in medicine, psychotherapy, and law. As a result, LLMs are widely promoted as accurate decision-support tools for well-structured tasks, and many users use them for this purpose [37,40-45]. However, when existing models are evaluated on real-world datasets, which more accurately reflect the complexity and ambiguity in real decision-making, their performance seems to be considerably worse [2,35,46].

The distinction between decision-making in idealized situations and in complex, ill-defined real-world settings has long been recognized in behavioral economics and psychology. In 1955, Herbert Simon introduced the concept of bounded rationality to study how human decision-making takes place under limited cognitive and environmental resources rather than under the conditions of perfect knowledge and unlimited resources, the normative ideal of full rationality, which is currently assumed in many LLM benchmarks [47,48]. Building on the idea of bounded rationality, the field of naturalistic decision-making (NDM) developed to study how experts make good decisions in real-world contexts [34,49]. Research in NDM shows that, rather than exhaustively comparing all possible options, both experts and novices typically rely on a limited set of information to recognize the most promising option or action [34,50,51]. This strategy is not perfectly accurate in all situations, but it often results in highly accurate decisions within short timeframes [50,52,53]. Specifically, findings from the NDM field suggest that in situations with high information validity, experts often perform on par with complex algorithms, even when using less information and simpler strategies [31,54,55]. This can be explained by the fact that experts rarely follow a strictly deductive or inductive process. Instead, they quickly recognize which information is relevant and engage in *abductive reasoning*; that is, they generate an initial hypothesis based on the observed piece of relevant information and then seek out information to test this hypothesis and update it as new contradicting information becomes available [28,56].

To describe human decision-making in real-world scenarios, 2 models feature most prominently in the NDM literature: recognition-primed decision-making (RPD) [56] and the data-frame theory [57]. The RPD model, which is used to make quick decisions in familiar situations, consists of 2 core processes: a pattern-matching loop and a mental simulation loop [56]. In the pattern matching loop, decision-makers assess whether a situation is familiar (eg, they recognize whether they have experienced a similar situation before). If the situation is recognized as familiar, they directly implement an action. If it is unfamiliar, they either reassess the situation or seek additional information until they achieve some sense of familiarity and can proceed to the mental simulation loop. In the subsequent mental simulation loop, decision-makers simulate implementing their chosen course of action. If they conclude that this action will most likely work, they implement it; if not, they modify the plan and reassess, or they consider new actions entirely [56].

The data-frame theory focuses more on sense-making and understanding new, unknown situations rather than on making decisions [57]. According to this model, humans use frames (basic ideas, hypotheses, or mental models about what is happening in a given context) and data (information in the environment). These 2 concepts interact: frames determine what information is noticed, sought, and how it is interpreted. At the same time, new data can lead a person to elaborate, revise, or even abandon their current frame. For example, in medical diagnosis, a physician may form an initial hypothesis (frame) based on presenting symptoms, then gather additional data to either confirm or reconsider that frame based on new information [57].

Although there is strong evidence supporting the occurrence, efficiency, and effectiveness of NDM models, such as the RPD model or the data-frame theory in real-world decision-making, these approaches have not yet been applied to instruct LLMs [58,59]. Existing reasoning prompts and models are inspired by an ideal form of human decision-making and deductive reasoning, and they seem to perform well on well-structured problems with known risks and gold-standard solutions, and less so in situations involving real-world ambiguity and uncertainty. Although for the latter situation, NDM-based strategies may prove more effective, they have not yet been applied to improve and evaluate LLM performance in ill-defined, real-world tasks. In this study, we aimed to test whether prompts based on NDM principles can improve LLM performance on a real-world, ill-defined task.

Building on our previous work, we used a standardized and validated evaluation framework that used a care-seeking or “self-triage” decision scenario involving real patient cases to evaluate NDM-based prompts [60]. Self-triage refers to the decision-making process by which people determine whether medical care is needed and, if so, where and how urgently to seek it (eg, self-care, primary care, or emergency care) [6,61,62]. This is a common decision task in everyday life, with 80% to 90% of the population reporting at least 1 symptom within a given month [63,64], and laypeople are increasingly consulting digital tools, such as LLMs, for advice [65,66]. Previous research shows that human performance in self-triage decisions is moderate and that LLMs perform only slightly better on average, although they almost always recommend professional care rather than self-care [6,67-69]. Self-triage is thus a suitable use case for the present study because it is typically ill-structured: information about symptoms may be incomplete and ambiguous, and decision-makers must decide under uncertainty. Therefore, self-triage is a representative example of the real-world decisions studied in NDM research.

We hypothesized that prompts inspired by the RPD model and the data-frame theory will significantly increase accuracy on these tasks in selecting the best course of action across both nonreasoning and reasoning models, compared to a standard zero-shot prompt.

## Methods

### Study Design

This evaluation study was designed as a prospective, longitudinal, observational LLM validation study. The intervention was the specific prompting strategy: a regular prompt, a recognition-primed prompt, and a data-frame prompt. We used these 3 prompting strategies to assess 45 vignettes across 10 models, each tested 10 times. The primary outcome was the accuracy of the models under each prompting condition, and the secondary outcome was the output variability of the tested models. No participants were involved in this study.

### Ethical Considerations

This study did not involve any prospective recruitment, interaction, or intervention with human participants. The LLM evaluation used an existing dataset of symptom descriptions originally collected on an online platform. Ethical approval for this collection, use, and deidentification of the cases was obtained from the ethics committee of the Department of Psychology and Ergonomics at Technische Universität Berlin (AWB_KOP_2_230711). For the present study, we accessed only the deidentified version of these cases [70]. Pseudonymized identifiers (eg, user names) were completely removed, and potential quasi-identifiers in free text (eg, city or institution names) were deleted. The dataset was stored on an access-restricted institutional computer, and the data were used solely for model evaluation. No attempts were made to reidentify individuals. Accordingly, no additional ethical approval was required for this secondary analysis. The reporting of this manuscript follows the TRIPOD (Transparent Reporting of a Multivariable Model for Individual Prognosis or Diagnosis)–LLM guideline [71].

### Tested Models

Because ChatGPT remains the most widely used LLM family [72], we focused our evaluation on LLMs currently available within ChatGPT. For the initial data collection, this included GPT-4o, GPT-4.1, GPT-4.1 mini, o3, o4 mini, and o4 mini high. For a second round of data collection, this included GPT-5.1 and GPT-5.2, including both the Instant and Thinking versions. All models are based on the Transformer architecture; however, o3, o4 mini, o4 mini high, GPT-5.1 Thinking, and GPT-5.2 Thinking include a reasoning process prior to generating output for users [23]. All models were tested using the default parameters to approximate consumer-facing ChatGPT model behavior. Thus, we used each model’s default temperature (1), a top-p of 1, did not specify a maximum output length (max_tokens unset), and did not specify a random seed. Because outputs are stochastic without a seed, we repeated each vignette-model-prompt condition 10 times and reported variability to approximate consumer-facing behavior. For GPT-5.1 and GPT-5.2, we set the reasoning.effort parameter to none (to disable reasoning and emulate consumer-facing GPT-5.1 and GPT-5.2 Instant), and to medium (to emulate GPT-5.1 and GPT-5.2 Thinking). Additionally, we conducted a sensitivity analysis using the 2 models for which the prompts yielded the largest accuracy gains. We tested a temperature of 0 (maximum determinism/control) and did not include a higher temperature option because the models frequently refused to provide recommendations with a temperature higher than the default temperature.

### Task and Evaluation Dataset

Our task consisted of obtaining advice on which care-seeking option is most appropriate for the described symptoms. This type of task is commonly used to evaluate both digital health applications and LLMs [6,61,62,73-75]. We selected it specifically because it reflects a real use case for ChatGPT [68,76-80], involves uncertainty (ie, unknown risks), and often deals with ambiguous or incomplete data or symptoms. For these reasons, we considered this task well suited to test the influence of NDM-inspired prompts on LLM performance in real-world problems.

The dataset was developed in previous studies and followed current guidelines for evaluating care-seeking decision support systems [70,81-83]. From an “ask the doctor” online platform, 45 real patient cases, where medical laypeople described their symptoms and sought advice from professionals, were collected between October 2023 and January 2024 and psychometrically validated [70,81]. Cases were further stratified to reflect the natural base rates of symptom types that are typically entered into online care-seeking advice tools based on the Centers for Disease Control and Prevention’s National Ambulatory Medical Care Survey [70,84]. Because of this stratification, the dataset included only 2 emergency care cases, 30 nonemergency care cases, and 13 self-care cases. To minimize editing effects, only typos were corrected. The original cases cannot be reproduced in this manuscript for copyright reasons, but they are available from the authors upon reasonable request. The cases describe acute symptoms for which laypeople seek decision support on whether and where to seek care. They cover a range of physical symptom presentations across specialties and are written in natural, nontechnical language from the perspective of medical laypeople. For example, 1 case describes a person reporting pain near the chest or lung area during the night when lying down, which improves when upright but worsens with breathing. After a few minutes, the person noticed a weird sensation, and the pain went away. The person then asks whether they should still seek medical care. Another exemplary case describes a person who notices a visible dent in the lower leg or heel area after sitting with one leg crossed for about 20 minutes. The person says this happens often and is usually painless, but this time the legs seem more swollen than usual, so the dent lasts longer.

To determine the most appropriate level of care, a physician panel consisting of 2 licensed physicians independently rated each case and then discussed their assessments until they reached a consensus assignment on 3 possible care-seeking options: (1) emergency care, defined as situations requiring emergency department visits; (2) nonemergency care, where consulting a general practitioner or another medical specialist is appropriate; and (3) self-care, where patients can monitor or manage symptoms themselves. Further details on the dataset are available in our publication on the RepVig framework [70].

Since the prompts included a specific output format, we used text pattern–based matching to extract the recommendation given by the LLM. If no direct match was found, we assessed the cases manually.

### Prompts

To evaluate the baseline accuracy of the tested models, we used the prompt shown in Textbox 1. This prompt was developed in previous studies evaluating the care-seeking advice of LLMs [67,69,70,85] and was adapted to specify an output structure (ie, “Answer: Classification”) that enabled us to classify model responses using natural language processing.

The NDM-inspired prompts were based on this base prompt but additionally included the name of the respective model (ie, “Recognition-Primed Decision-Making model” and “Data-Frame-Theory approach”) to prime the LLMs accordingly. Each prompt then provided instructions on how to reason following the principles of the corresponding model. The full prompts are shown in Textbox 2.

Textbox 1. **Care-seeking advice prompt used in the control group to determine the base accuracy of the tested large language models.**

Please give a triage classification for the following vignette. The criteria for triage classifications are presented below:

- Emergency: Call 911 or go directly to the emergency room;
- Nonemergency: Try to see a doctor, but the symptoms do not require immediate attention at an emergency room. For example, you can see a doctor in the next week.
- Self-care: Let the health issue get better on its own and review the situation again in a few days.

Respond in the following format:Answer: Your answer [either Emergency, Nonemergency, or Self-Care].

Textbox 2. **Recognition-primed prompt instructing the large language models to reason according to the recognition-primed decision-making model.**

Please give a triage classification for the following vignette. The criteria for triage classifications are presented below:

- Emergency: Call 911 or go directly to the emergency room;
- Nonemergency: Try to see a doctor, but the symptoms do not require immediate attention at an emergency room. For example, you can see a doctor in the next week.
- Self-care: Let the health issue get better on its own and review the situation again in a few days.

Use the recognition-primed decision-making model to make your decision. Does this situation match any typical cases you know? If yes, what is the usual decision for such a case? Simulate implementing this decision for the described situation and test whether it will work. If not, modify it and test again whether it will work. If the situation is not similar to any typical case you know, try to reassess the situation until you think it sounds familiar, simulate the implementation again, and test whether it will work.

Respond in the following format:Analysis: Your analysis || Answer: Your answer [either Emergency, Nonemergency, or Self-Care].

Textbox 3. **Data-frame prompt instructing the large language models to reason according to the data-frame theory.**

Please give a triage classification for the following vignette. The criteria for triage classifications are presented below:

- Emergency: Call 911 or go directly to the emergency room;
- Nonemergency: Try to see a doctor, but the symptoms do not require immediate attention at an emergency room. For example, you can see a doctor in the next week.
- Self-care: Let the health issue get better on its own and review the situation again in a few days.

Before giving your triage classification, think about the correct classification using the Data-Frame-Theory approach. As you analyze the vignette, actively use the following reasoning processes (as needed, not necessarily in order):

- Construct or recognize a frame: Identify the main interpretation or mental model that organizes the case information.
- Elaborate the frame: Seek out or infer additional relevant details from the vignette.
- Question the frame: Look for inconsistencies, surprising data, or violated expectations.
- Preserve the frame: Consider whether your interpretation still fits, or if any data needs to be reinterpreted.
- Seek a new frame: If appropriate, consider alternative interpretations.
- Reframe: Revise your perspective and reinterpret the data if needed.
- Compare frames: Identify and weigh alternative ways of understanding the case.

Respond in the following format:Reflection process: Your reflection || Answer: Your answer [either Emergency, Nonemergency, or Self-Care].

All prompts were tested for feasibility in a pretest, during which the authors tested the prompts and API calls using random cases and manually assessed the output for adherence to the instructions and correct formatting.

### Procedure

We used a custom-built Python script to access the OpenAI API on May 23, 2025, and a second time for newer models on February 23, 2026. For each model, the prompts (Textboxes 1-3) were entered as system prompts, and the case vignettes as user prompts. The context window was cleared before every call. Because of the high output variability observed in LLMs [67,86,87], we tested each model on each case 10 times as a quality-management measure to account for the fact that different users may receive different advice for the same input. Model outputs were then classified automatically in R into 3 categories (emergency, nonemergency, self-care). For cases in which the category could not be determined through keyword or pattern matching (n=61), manual coding was performed by reading through the answer and assigning a classification manually.

### Outcome Measures

The primary outcome was classification accuracy, defined as whether the model’s triage recommendation matched the physician-panel gold standard reference for each vignette (ie, correct or incorrect). This metric was chosen because it most closely measures the potential behavioral and safety impact the prompts can have on users. Secondary outcomes included the accuracy by each triage level (emergency, nonemergency, or self-care), calculated as the proportion of correct recommendations within each stratum. Additionally, because the vignette set included only 2 emergency cases, we dichotomized the triage levels into 2 groups: requiring medical care versus self-care. Next, we assessed output variability using Fleiss’ Kappa for each model-vignette-prompt combination, and by assessing the consistency of model recommendations, that is, the proportion of the 10 trials corresponding to the most frequently given recommendation. Lastly, we assessed technical accuracy by coding whether the correct recommendation was given at least once among all 10 trials.

### Data Analysis

All analyses were conducted in R using the packages symptomcheckR, tidyverse, psych, and lme4 [88-91]. To assess the accuracy of each prompt, we calculated the mean proportion of correctly solved cases and quantified precision using 95% CIs. To test our hypothesis that the NDM-inspired prompts increase LLM performance, we used mixed effects binomial logistic regression (with prompt type as a fixed effect and random intercepts for model, vignette, and model-by-vignette combination to account for repeated observations and clustering in our data—that is, for having each model assess each vignette 10 times) with 2-sided tests. Additionally, we conducted subgroup analyses to assess accuracy in dichotomized decisions (ie, professional care vs self-care), accuracy by model, and accuracy by each care-seeking level. In sensitivity analyses, we further tested whether the reported results remained stable with a low-temperature setting. We chose the 2 models with the highest and lowest prompt-dependent accuracy improvement (ie, GPT-4.1 mini and GPT-5.2 Instant).

To quantify output variability for each prompt, vignette, and model combination, we calculated Fleiss κ and recorded the frequency with which the most common recommendation was given across the 10 trials for each vignette and model. As an estimate of “technical accuracy” (ie, whether the model was technically capable of generating the correct advice), we noted whether the correct recommendation was given at least once in 10 trials [67,92].

## Results

### Assessments

We used 45 vignettes to test 10 models, with each model run 10 times per vignette using 3 prompting strategies (default, recognition-primed prompting, and data-frame prompting). This resulted in a total of 13,500 individual assessments.

### Overall Accuracy of Each Prompt

The average accuracy across all models, vignettes, and trials was 63.3% (95% CI 61.9%‐64.7%) for the default prompt, 67.6% (95% CI 66.3%‐69%) for the recognition-primed prompt, and 66.7% (95% CI 65.3%‐68%) for the data-frame prompt. Both the recognition-primed prompt (OR 2.26, *z*=8.69; *P*<.001) and the data-frame prompt (OR 2.05, *z*=7.23; *P*<.001) significantly increased accuracy compared to the default prompt. Improvements were greater for reasoning models than for nonreasoning models (OR 2.15, *z*=4.05, *P*<.001 for the recognition-primed prompt and OR 1.70, *z*=2.65, *P*=.008 for the data-frame prompt). The largest increase in accuracy compared to the default prompt was observed for GPT-4.1 mini with the data-frame prompt, with an improvement of 13 percentage points (95% CI 9.7‐16.1), as shown in Table 1. In a binary choice, that is, care versus self-care, results remained similar, as shown in Table S1 in Multimedia Appendix 1. The same holds true when tested with a low-temperature setting, as shown in Table S2 in Multimedia Appendix 1.

**Table 1.** The accuracy of all tested models for each prompt.

Model	Default prompt, mean (95% CI)	Recognition-primed prompt, mean (95% CI)	Data-frame prompt, mean (95% CI)	Model type
Overall (%)	63.3 (61.9‐64.7)	67.6 (66.3 ‐69)	66.7 (65.3‐68)	—^a^
GPT-4o (%)	65.3 (60.8‐69.6)	70.7 (66.3‐74.7)	66.2 (61.7‐70.4)	Nonreasoning
GPT-4.1 (%)	64.4 (59.9‐68.7)	72.7 (68.4‐76.6)	72.4 (68.1‐76.4)	Nonreasoning
GPT-4.1 mini (%)	49.8 (45.2‐54.4)	60.7 (56.1‐65.1)	62.4 (57.9‐66.8)	Nonreasoning
o3 (%)	70.7 (66.3‐74.7)	75.1 (70.9‐78.9)	76.2 (72.1‐79.9)	Reasoning
o4 mini (%)	69.3 (64.9‐73.4)	70.7 (66.3‐74.7)	72.9 (68.6‐76.8)	Reasoning
o4 mini high (%)	68.9 (64.5‐73)	71.3 (67‐75.3)	70.7 (66.3‐74.7)	Reasoning
GPT-5.1 Instant (%)	64 (59.4‐68.4)	71.1 (66.7‐75.3)	66.2 (61.6‐70.6)	Nonreasoning
GPT-5.1 Thinking (%)	70.7 (66.2‐74.8)	74.7 (70.4‐78.6)	72.4 (68.1‐76.5)	Reasoning
GPT-5.2 Instant (%)	57.8 (53.1‐62.4)	56.4 (51.7‐61.1)	55.3 (50.6‐60)	Nonreasoning
GPT-5.2 Thinking (%)	52 (47.3‐56.7)	53.1 (48.4‐57.8)	51.8 (47.1‐56.5)	Reasoning

a Not available.

The recognition-primed prompt increased accuracy in 18 of 45 cases (40%) and decreased accuracy in 13 cases (29%) on average across all models. Its median increase in accuracy was 18% (IQR 5%‐24%), whereas the median decrease was 5% (IQR 2%‐7%). The data-frame prompt increased accuracy in 17 of 45 (38%) cases and reduced it in 12 (27%) cases. Its median increase in accuracy was 14% (IQR 4%‐19%), and the median decrease was 5% (IQR 3%‐8%). Most decreases in accuracy affected nonemergency cases (12/13, 92% for the recognition-primed prompt and 11/12, 92% for the data-frame prompt, see the Triage-Level Accuracy of Each Prompt section for the direction). Increases were observed in both nonemergency and self-care cases (8/18, 44%, and 10/18, 56%, respectively, for the recognition-primed prompt; 8/17, 47%, and 9/17, 53%, for the data-frame prompt, see the next section for the direction) (Table S3 in Multimedia Appendix 1).

### Triage-Level Accuracy of Each Prompt

**Figure 1.**
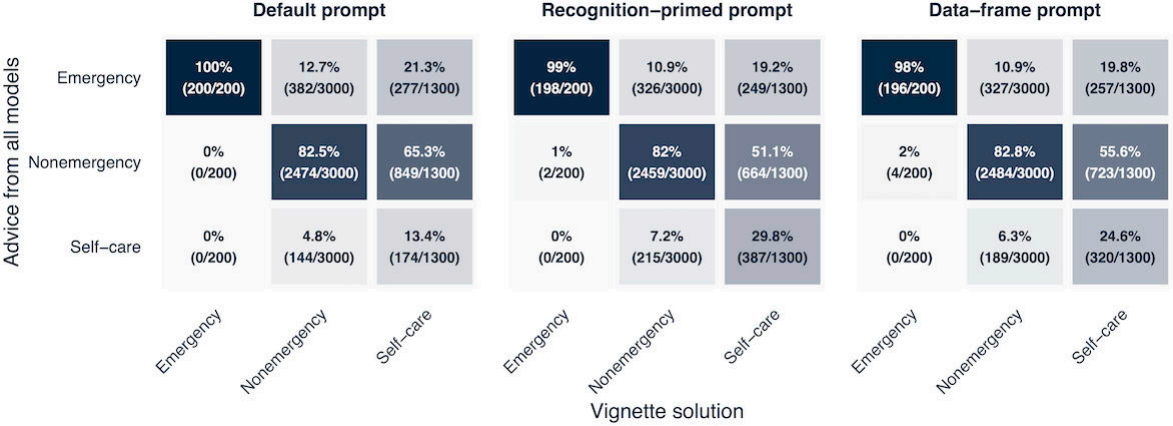
Confusion matrix showing the classification of each prompt across all models compared to the correct vignette solution. Emergency estimates may be unreliable because only 2 cases were included.

Across all 3 prompts, the models tended to recommend higher-than-necessary urgency (accounting for 88% of all errors, 95% CI 87%‐88.9%) rather than lower-than-necessary urgency (12% of all errors, 95% CI 11.1%‐13%). With the default prompt, both emergency cases were correctly identified (100%, 95% CI 98.2%‐100%). Using both the data-frame prompt and the recognition-primed prompt, both emergency cases were also mostly identified correctly (99%, 95% CI 96.4%‐99.9% and 98%, 95% CI 95%‐99.5%, respectively), although some trials resulted in incorrect nonemergency advice (1%, 95% CI 0.1%‐3.6% and 2%, 95% CI 0.5%‐5%, respectively). Accuracy for nonemergency cases was similar across all prompts: 82.5% (95% CI 81.1%‐83.8%) for the default prompt, 82% (95% CI 80.5%‐83.3%) for the recognition-primed prompt, and 82.8% (95% CI 81.4%‐84.1%) for the data-frame prompt. The largest difference was observed for self-care cases: With the default prompt, the models correctly identified only 13.4% (95% CI 11.6%‐15.4%), compared to 29.8% (95% CI 27.3%‐32.3%) with the recognition-primed prompt and 24.6% (95% CI 22.3%‐27.1%) with the data-frame prompt (Figure 1). The results remained similar in a binary choice task and also when tested with a low temperature setting (Tables S1 and S4 in Multimedia Appendix 1).

Notably, nonreasoning models that never or rarely provided self-care advice with the default prompt began providing self-care advice with relatively high accuracy when using the NDM-inspired prompts (eg, 0%, 95% CI 0%‐2.9% for the default prompt in GPT-4.1, compared to 43.8%, 95% CI 35.6%‐52.4% for the recognition-primed prompt and 39.2%, 95% CI 31.3%‐47.8% for the data-frame prompt). For reasoning models, which already gave self-care advice with the default prompt, accuracy further improved with the NDM-inspired prompts (eg, 46.9%, 95% CI 38.6%‐55.5% with the default prompt in o4 mini; 63.8%, 95% CI 55.3%‐71.6% with the recognition-primed prompt; and 56.2%, 95% CI 47.6%‐64.4% with the data-frame prompt; Table 2).

**Table 2.** Accuracy of each model and prompt in generating care-seeking advice by correct vignette solution.

Model and vignette type	Default prompt, mean (95% CI)	Recognition-primed prompt, mean (95% CI)	Data-frame prompt, mean (95% CI)
GPT-4o (%)			
Emergency (n=2)	100 (83.9‐100)	100 (83.9‐100)	100 (83.9‐100)
Nonemergency	90.3 (86.5‐93.2)	92.3 (88.8‐94.8)	87.7 (83.5‐90.9)
Self-care	2.3 (0.8‐6.6)	16.2 (10.8‐23.4)	11.5 (7.1‐18.2)
GPT-4.1 (%)			
Emergency (n=2)	100 (83.9‐100)	100 (83.9‐100)	80 (58.4‐91.9)
Nonemergency	90 (86.1‐92.9)	83.3 (78.7‐87.1)	86.3 (82‐89.8)
Self-care	0 (0‐2.9)	43.8 (35.6‐52.4)	39.2 (31.3‐47.8)
GPT-4.1 mini (%)			
Emergency (n=2)	100 (83.9‐100)	100 (83.9‐100)	100 (83.9‐100)
Nonemergency	68 (62.5‐73)	81.3 (76.5‐85.3)	87 (82.7‐90.3)
Self-Care	0 (0‐2.9)	6.9 (3.7‐12.6)	0 (0‐2.9)
o3 (%)			
Emergency (n=2)	100 (83.9‐100)	100 (83.9‐100)	100 (83.9‐100)
Nonemergency	93.7 (90.3‐95.9)	90.7 (86.8‐93.5)	92.7 (89.1‐95.1)
Self-care	13.1 (8.3‐19.9)	35.4 (27.7‐43.9)	34.6 (27‐43.1)
o4 mini (%)			
Emergency (n=2)	100 (83.9‐100)	100 (83.9‐100)	100 (83.9‐100)
Nonemergency	77 (71.9‐81.4)	71.7 (66.3‐76.5)	78.3 (73.3‐82.6)
Self-care	46.9 (38.6‐55.5)	63.8 (55.3‐71.6)	56.2 (47.6‐64.4)
o4 mini high (%)			
Emergency (n=2)	100 (83.9‐100)	100 (83.9‐100)	100 (83.9‐100)
Nonemergency	75.7 (70.5‐80.2)	74.7 (69.5‐79.3)	74 (68.8‐78.6)
Self-care	48.5 (40‐57)	59.2 (50.6‐67.3)	58.5 (49.9‐66.6)
GPT-5.1 Instant (%)			
Emergency (n=2)	100 (83.9‐100)	95 (76.4‐99.1)	100 (83.9‐100)
Nonemergency	87 (82.7–90.3)	86 (81.6‐89.5)	88 (83.8‐91.2)
Self-care	5.4 (2.6‐10.7)	33.1 (25.6‐41.5)	10.8 (6.5‐17.3)
GPT-5.1 Thinking (%)			
Emergency (n=2)	100 (83.9‐100)	95 (76.4‐99.1)	100% (83.9%‐100%)
Nonemergency	92 (88.4‐94.6)	90 (86.1‐92.9)	87 (82.7‐90.3)
Self-care	16.9 (11.4‐24.3)	36.2 (28.4‐44.7)	34.6 (27‐43.1)
GPT-5.2 Instant (%)			
Emergency (n=2)	100 (83.9‐100)	100 (83.9‐100)	100 (83.9‐100)
Nonemergency	80 (75.1‐84.1)	76.7 (71.6‐81.1)	76.3 (71.2‐80.8)
Self-care	0 (0‐2.9)	3.1 (1.2‐7.6)	0 (0‐2.9)
GPT-5.2 Thinking (%)			
Emergency (n=2)	100 (83.9‐100)	100 (83.9‐100)	100 (83.9‐100)
Nonemergency	71 (65.6‐75.8)	73 (67.7‐77.7)	70.7 (65.3‐75.5)
Self-care	0.8 (0.1‐4.2)	0 (0‐2.9)	0.8 (0.1‐4.2)

### Output Variability of Each Prompting Technique

Intertrial reliability—that is, the frequency with which a vignette received the same advice from the same model across multiple trials—was comparable across all prompts, with median Fleiss κ values of 0.766 (IQR 0.706‐0.884) for the default prompt, 0.717 (IQR 0.681‐0.743) for the recognition-primed prompt, and 0.751 (IQR 0.725‐0.773) for the data-frame prompt (Table 3). The results remained similar when tested with a low temperature setting (Table S5 in Multimedia Appendix 1).

**Table 3.** Intertrial reliability (Fleiss κ) of each model for each prompt.

Model	Default prompt	Recognition-primed prompt	Data-frame prompt
Overall, median (IQR)	0.766 (0.706‐0.884)	0.717 (0.681‐0.743)	0.751 (0.725‐0.773)
GPT-4o	0.708	0.710	0.718
GPT-4.1	0.963	0.777	0.658
GPT-4.1 mini	0.824	0.634	0.756
o3	0.706	0.675	0.807
o4 mini	0.707	0.699	0.766
o4 mini high	0.701	0.743	0.744
GPT-5.1 Instant	0.895	0.644	0.620
GPT-5.1 Thinking	0.689	0.723	0.745
GPT-5.2 Instant	0.926	0.742	0.775
GPT-5.2 Thinking	0.851	0.821	0.839

**Figure 2.**
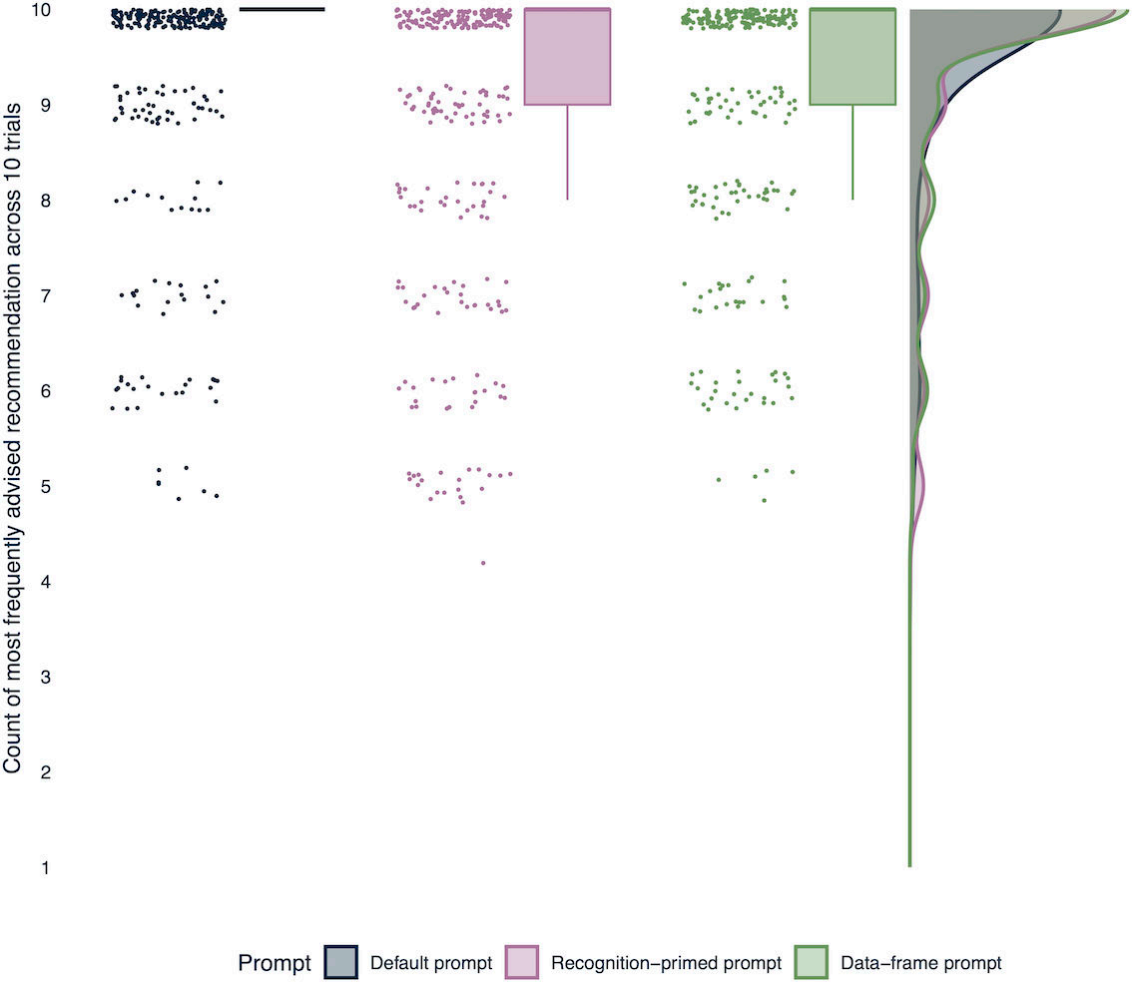
Number of times the most frequently advised recommendation was given among the 3 prompts.

When considering the most frequently given recommendation for each vignette by each model, all prompts yielded relatively consistent advice across multiple trials (mean 76.9%, 95% CI 72.7%‐80.7% for the default prompt; mean 66.9%, 95% CI 62.3%‐71.2% for the recognition-primed prompt; mean 71.1%, 95% CI 66.7%‐75.3% for the data-frame prompt), as shown in Figure 2. There was no statistically significant difference between the prompts in how often a specific option was recommended across trials (*z*=1.50, *P*=.13 for the recognition-primed prompt; *z*=0.72, *P*=.47 for the data-frame prompt).

However, the tested models were more likely to provide the correct solution at least once across multiple trials when using the recognition-primed prompt (mean 82.2%, 95% CI 78.4%‐85.6%) and the data-frame prompt (mean 78.4%, 95% CI 74.4%‐82.2%) compared to the default prompt (mean 73.1%, 95% CI 68.8%‐77.2%) (Table 4). The results remained similar when tested with a low-temperature setting (Table S6 in Multimedia Appendix 1).

**Table 4.** Percentage of cases that were solved correctly at least once among 10 trials.

Model	Default prompt, mean (95% CI)	Recognition-primed prompt, mean (95% CI)	Data-frame prompt, mean (95% CI)
Overall (%)	73.1 (68.8‐77.2)	82.2 (78.4‐85.6)	78.4 (74.4‐82.2)
GPT-4o (%)	77.8 (62.9‐88.8)	84.4 (70.5‐93.5)	80 (65.4‐90.4)
GPT-4.1 (%)	66.7 (51‐80)	84.4 (70.5‐93.5)	84.4 (70.5‐93.5)
GPT-4.1 mini (%)	55.6 (40‐70.4)	75.6 (60.5‐87.1)	66.7 (51‐80)
o3 (%)	82.2 (67.9‐92)	91.1 (78.8‐97.5)	84.4 (70.5‐93.5)
o4 mini (%)	86.7 (73.2‐94.9)	88.9 (75.9‐96.3)	86.7 (73.2‐94.9)
o4 mini high (%)	91.1 (78.8‐97.5)	88.9 (75.9‐96.3)	86.7 (73.2‐94.9)
GPT-5.1 Instant (%)	68.9 (53.4‐81.8)	86.7 (73.2‐94.9)	82.2 (67.9‐92)
GPT-5.1 Thinking (%)	82.2 (67.9‐92)	88.9 (75.9‐96.3)	86.7 (73.2‐94.9)
GPT-5.2 Instant (%)	60 (44.3‐74.3)	73.3 (58.1‐85.4)	64.4 (48.8‐78.1)
GPT-5.2 Thinking (%)	60 (44.3‐74.3)	60 (44.3‐74.3)	62.2 (46.5‐76.2)

## Discussion

### Principal Results

Our study investigated whether prompting strategies inspired by NDM—a field that analyzes how humans make real-world decisions under uncertainty—can improve LLM performance in ill-defined tasks such as care-seeking decisions. Our results show that both the recognition-primed and the data-frame prompts increased the accuracy of care-seeking advice across all tested models except GPT-5.2. Although this effect may partly reflect the additional reasoning process before producing an answer among nonreasoning models, we observed improvements not only in nonreasoning models but also in reasoning models that already include a reasoning process. This observation suggests that our results cannot simply be attributed to a general reasoning process. Notably, most nonreasoning models with NDM-inspired prompts outperformed traditional reasoning models using the default prompt, and reasoning models also showed significant improvements with the NDM-inspired prompts.

The greatest improvements due to the NDM-inspired prompts were seen in self-care cases, which were more often correctly identified. Nonreasoning models rarely or never provided self-care advice with the default prompt, a finding consistent with previous studies [67,69,70]. When prompted with the NDM-inspired prompts, these models began giving self-care advice and even reached a relatively high level of accuracy, up to 44%. In contrast, accuracy for the 2 included emergency cases and the included nonemergency cases showed little change, likely due to a ceiling effect, as the tested models were already highly accurate on these cases with the default prompt. Prior research suggests that self-care advice is typically given by LLMs only when a reasoning process is included and that the tendency toward risk-averse recommendations may stem from built-in safety measures [67,69]. The recognition-primed prompt explicitly instructs the model to recall similar situations (pattern matching according to the RPD model) and to forecast possible outcomes (mental simulation), which may help the model reconsider overly cautious recommendations before giving advice. Similarly, the data-frame prompt encourages the model to re-examine each initial recommendation and—if new data do not fit the initial frame—explore alternative frames, which may help identify when self-care is sufficient rather than defaulting to medical referral.

OpenAI’s most recent GPT-5 model family includes an updated reasoning process [26,27]. The benefits of using NDM-inspired prompts were replicated for GPT-5.1 (for both the Instant version without a reasoning process and the Thinking version with a reasoning process), but not for GPT-5.2: self-care accuracy dropped to 0% in both GPT-5.2 Instant and GPT-5.2 Thinking and remained unchanged with NDM-inspired prompts. These results may suggest a version-level shift toward recommending professional care that prompting does not alter. This observation is unlikely to be attributable to changes in the reasoning mechanism alone, because GPT-5.1—despite also using the updated reasoning process—did not show the same decrease in self-care accuracy.

### Implications

The present findings have implications for prompt engineering, artificial intelligence (AI) research, and end users. First, for prompt engineering, we suggest that, rather than relying solely on prompts built on computer science (eg, ensemble methods and decomposing), strategies derived from cognitive science, applied psychology, and HF/E—especially those based on models of human decision-making under uncertainty—may be more effective or, at least, serious competitors, particularly in domains with high ambiguity and uncertainty, such as triage or diagnostic decisions. In these ill-defined situations, we showed that a “reasoning blueprint” based on human cognition can outperform methods that simply instruct the models to reason. We acknowledge, however, that, based on our results, the benefits of NDM-inspired prompts are thus far limited to uncertain tasks. It remains to be seen how they perform on more well-defined tasks, such as text formatting or summarization.

### Limitations

Although our results show a positive impact of combining NDM with prompt engineering, there are several limitations. First, we conducted a single benchmarking test within one domain, that is, care-seeking advice. Although this is a typical real-world decision task with high uncertainty and a common use case for LLMs [68,76-80], it remains unclear whether our findings generalize to other tasks or domains with varying levels of ambiguity and/or uncertainty. Second, the low sample size for emergency cases leads to unstable accuracy estimates, and no safety conclusions should be derived from the data presented here. Third, we limited our evaluation to LLMs that are currently integrated into ChatGPT. We made this decision to assess practical impact for users; however, it is unclear whether these results would hold true for the broader range of LLMs available or in development. In particular, future research should test whether similar results can be achieved with smaller models and limited context windows, given that the reasoning process increases token requirements.

The NDM-inspired prompts themselves present another limitation. These prompts are computationally more expensive to run than standard user inputs because they add a reasoning output before giving advice. Although this may not affect individual users, it could increase operational costs for developers integrating such prompts, especially compared to nonreasoning models. We recommend that any potential performance gains from NDM-inspired prompting be carefully weighed against increased costs on a case-by-case basis.

Next, we did not include participants who interacted with the LLMs directly. Instead, we used a highly controlled setup in which each model was prompted repeatedly using standardized prompts. In real-world use, however, users’ prompts vary substantially in both content and quality [4,70,93]. Accordingly, the present study was designed to test whether NDM-inspired prompts can improve model accuracy under controlled conditions; we cannot infer that these prompts would translate into improved user decisions or higher-quality outputs in everyday use. This work should, therefore, be interpreted as a technical evaluation of model behavior under controlled inputs rather than as a clinical validation study. Depending on the intended use, LLMs may be regulated as Software as a Medical Device and may, therefore, require additional evidence that is outside the scope of the present study. Recent work on differing user inputs and adversarial attacks in chatbots shows that they can produce unsafe outputs depending on the specific prompts, which further demonstrates that more rigorous and use-case-specific safety evaluations are needed before deployment [94,95]. Future studies should therefore conduct user studies to examine whether NDM-inspired prompts also yield better recommendations and decision support for users in real-world settings, and to determine how NDM-inspired prompts may be used to prevent adversarial attacks.

Finally, the prompts tested here were based on only 2 decision-making models. There are other models that could serve as inspiration for prompt development, such as the decision ladder or heuristic decision models [51,96]. Moreover, domain-specific decision-making models may be even better suited for certain use cases. For care-seeking advice, no such model currently exists to explain how humans make these decisions. However, the development of such a model could be helpful to develop even more targeted prompting strategies to further increase LLM performance.

### Future Research

This study is among the first to combine NDM and AI-based decision support systems to foster more naturalistic decision support. Our findings provide a foundation for future work by demonstrating that real-world human reasoning strategies can improve the accuracy of LLMs. Building on these results, future work could examine how NDM and AI can be combined to support users. For example, prompting LLMs to use reasoning processes that reflect human decision-making could open a new direction for explainable AI. Unlike traditional explainable AI methods that focus on feature importance, providing explanations based on human-like pattern recognition and mental simulation may increase trust and help users identify potential mistakes in the reasoning process. Prior research has shown that users critically assess, rather than blindly follow, AI advice [4]. Giving users an NDM-inspired reasoning approach may support this evaluation more than providing advice with a post hoc explanation.

NDM-inspired prompts may also improve human-AI collaboration: When humans and AI share a conceptual language (consisting of frames, pattern matching, and mental simulation), it may become easier for users to integrate AI advice into their own reasoning. For example, physicians could review the frames used by the LLM, add new data points, and let the AI simulate whether these fit the frame. Conversely, the AI could make predictions based on its frame, which the physician can cross-check with clinical data. An AI would thus not only give a final recommendation but also provide support in hypothesis generation, data gathering, and hypothesis testing [97,98].

Next, NDM-inspired prompting could also be used for education and training. LLMs could serve as interactive tools for medical students, allowing them to practice decision-making using the RPD model alongside the AI by comparing their mental simulations with those of the model. The AI could then provide feedback on differences in their respective frames.

More broadly, future work should move beyond technical benchmarking toward evaluation designs aligned with Software as a Medical Device expectations by predefining the intended use case and testing performance and safety prospectively in real-world settings. In this context, NDM may be treated as a theoretical basis for uncertainty management, and future studies can test whether NDM-based prompts reduce failures across different user inputs and adversarial attacks.

### Conclusions

In this study, we showed that applying models from NDM to prompt LLMs can improve performance in highly uncertain and ambiguous care-seeking tasks. Both NDM-inspired prompts tested here increased overall accuracy across both reasoning and nonreasoning models, with the greatest improvement in self-care recommendations, while maintaining high accuracy in the 2 included emergency cases and all included nonemergency cases. These findings may open up a new strategy for prompt engineering: rather than relying on prompts derived from computer science, prompts that build on NDM models or related models from applied psychology and HF/E, which represent how humans make sense of uncertainty, may be more effective in ill-defined tasks. As LLMs and other AI tools are increasingly adopted in safety-critical and everyday applications, NDM-inspired prompting may offer a strategy for making AI more useful for real-world decision-making.

## Supplementary material

10.2196/88053 Multimedia Appendix 1 Additional accuracy and sensitivity analyses.

## Abbreviations

AI: artificial intelligence
HF/E: human factors and ergonomics
LLM: large language model
NDM: naturalistic decision-making
RPD: recognition-primed decision-making
TRIPOD: Transparent Reporting of a Multivariable Model for Individual Prognosis or Diagnosis
