# Supplementary material for: Increasing Large Language Model Accuracy for Care-Seeking Advice Using Prompts Reflecting Human Reasoning Strategies in the Real World: Validation Study
Source: JMIR Biomed Eng. 2026 Apr 8;11:e88053. doi: 10.2196/88053 (PMC13061108; doi:10.2196/88053)
Supplement: Multimedia Appendix 1 [file biomedeng-v11-e88053-s001.docx]

Table S1. The accuracy, sensitivity, and specificity of each prompt in a dichotomized (i.e., care vs. self-care) decision.

|  | Accuracy Default Prompt | Accuracy Recognition-Primed Prompt | Accuracy Data/Frame-Prompt | Sensitivity Default Prompt | Sensitivity Recognition-Primed Prompt | Sensitivity Data/Frame-Prompt | Specificity Default Prompt | Specificity Recognition-Primed Prompt | Specificity Data/Frame-Prompt |
| --- | --- | --- | --- | --- | --- | --- | --- | --- | --- |
| Overall, Mean (95% CI) | 63.3% (61.9–64.7%) | 67.6% (66.3–69.0%) | 66.7% (65.3–68.0%) | 82.5% (81.1–83.8%) | 82.0% (80.5–83.3%) | 82.8% (81.4–84.1%) | 13.4% (11.6–15.4%) | 29.8% (27.3–32.3%) | 24.6% (22.3–27.1%) |
| GPT 4o, Mean (95% CI) | 65.3% (60.7–69.7%) | 70.7% (66.2–74.8%) | 66.2% (61.6–70.6%) | 90.3% (86.4–93.4%) | 92.3% (88.7–95.1%) | 87.7% (83.4–91.2%) | 2.3% (0.5–6.6%) | 16.2% (10.3–23.6%) | 11.5% (6.6–18.3%) |
| GPT 4.1, Mean (95% CI) | 64.4% (59.8–68.9%) | 72.7% (68.3–76.7%) | 72.4% (68.1–76.5%) | 90.0% (86.0–93.2%) | 83.3% (78.6–87.4%) | 86.3% (81.9–90.0%) | 0.0% (0.0–2.8%) | 43.8% (35.2–52.8%) | 39.2% (30.8–48.2%) |
| GPT 4.1 mini, Mean (95% CI) | 49.8% (45.1–54.5%) | 60.7% (56.0–65.2%) | 62.4% (57.8–66.9%) | 68.0% (62.4–73.2%) | 81.3% (76.5–85.6%) | 87.0% (82.7–90.6%) | 0.0% (0.0–2.8%) | 6.9% (3.2–12.7%) | 0.0% (0.0–2.8%) |
| o3, Mean (95% CI) | 70.7% (66.2–74.8%) | 75.1% (70.8–79.0%) | 76.2% (72.0–80.1%) | 93.7% (90.3–96.1%) | 90.7% (86.8–93.7%) | 92.7% (89.1–95.3%) | 13.1% (7.8–20.1%) | 35.4% (27.2–44.2%) | 34.6% (26.5–43.5%) |
| o4 mini, Mean (95% CI) | 69.3% (64.8–73.6%) | 70.7% (66.2–74.8%) | 72.9% (68.5–76.9%) | 77.0% (71.8–81.6%) | 71.7% (66.2–76.7%) | 78.3% (73.2–82.9%) | 46.9% (38.1–55.9%) | 63.8% (55.0–72.1%) | 56.2% (47.2–64.8%) |
| o4 mini high, Mean (95% CI) | 68.9% (64.4–73.1%) | 71.3% (66.9–75.5%) | 70.7% (66.2–74.8%) | 75.7% (70.4–80.4%) | 74.7% (69.3–79.5%) | 74.0% (68.6–78.9%) | 48.5% (39.6–57.4%) | 59.2% (50.3–67.8%) | 58.5% (49.5–67.0%) |
| GPT 5.1 Instant, Mean (95% CI) | 64.0% (59.4–68.4%) | 71.1% (66.7–75.3%) | 66.2% (61.6–70.6%) | 87.0% (82.7–90.6%) | 86.0% (81.6–89.7%) | 88.0% (83.8–91.5%) | 5.4% (2.2–10.8%) | 33.1% (25.1–41.9%) | 10.8% (6.0–17.4%) |
| GPT 5.1 Thinking, Mean (95% CI) | 70.7% (66.2–74.8%) | 74.7% (70.4–78.6%) | 72.4% (68.1–76.5%) | 92.0% (88.3–94.8%) | 90.0% (86.0–93.2%) | 87.0% (82.7–90.6%) | 16.9% (10.9–24.5%) | 36.2% (27.9–45.0%) | 34.6% (26.5–43.5%) |
| GPT 5.2 Instant, Mean (95% CI) | 57.8% (53.1–62.4%) | 56.4% (51.7–61.1%) | 55.3% (50.6–60.0%) | 80.0% (75.0–84.4%) | 76.7% (71.5–81.3%) | 76.3% (71.1–81.0%) | 0.0% (0.0–2.8%) | 3.1% (0.8–7.7%) | 0.0% (0.0–2.8%) |
| GPT 5.2 Thinking, Mean (95% CI) | 52.0% (47.3–56.7%) | 53.1% (48.4–57.8%) | 51.8% (47.1–56.5%) | 71.0% (65.5–76.1%) | 73.0% (67.6–77.9%) | 70.7% (65.2–75.8%) | 0.8% (0.0–4.2%) | 0.0% (0.0–2.8%) | 0.8% (0.0–4.2%) |

Table S2. Sensitivity Analysis: Two models and their accuracy across prompts with different temperature settings.

|  | Default Prompt, Default Temperature | Recognition-Primed Prompt, Default Temperature | Data/Frame-Prompt, Default Temperature | Default Prompt, Low Temperature | Recognition-Primed Prompt, Low Temperature | Data/Frame-Prompt, Low Temperature |
| --- | --- | --- | --- | --- | --- | --- |
| Overall, Mean (95% CI) | 53.8%  (50.5–57.1%) | 58.6%  (55.3–61.8%) | 58.9%  (55.6–62.1%) | 51.6%  (48.2–54.9%) | 58.4%  (55.1–61.7%) | 59.1%  (55.6–62.1%) |
| GPT 4.1 mini, Mean (95% CI) | 49.8%  (45.2–54.4%) | 60.7%  (56.1–65.1%) | 62.4%  (57.9–66.8%) | 48.0%  (43.3–52.7%) | 61.1%  (56.4–65.6%) | 63.6%  (58.9–68.0%) |
| GPT 5.2 Instant, Mean (95% CI) | 57.8% (53.1–62.4%) | 56.4% (51.7–61.1%) | 55.3% (50.6–60.0%) | 55.1% (50.4–59.8%) | 55.8% (51.1–60.4%) | 54.7% (49.9–59.3%) |

Table S3. The accuracy of each prompt when assessing each vignette.

| Vignette | Default Prompt | Recognition-Primed Prompt | Data/Frame-Prompt | Vignette Solution |
| --- | --- | --- | --- | --- |
| 1 | 37.0% (27.6–47.2%) | 63.0% (52.8–72.4%) | 52.0% (41.8–62.1%) | Self-Care |
| 2 | 4.0% (1.1–9.9%) | 8.0% (3.5–15.2%) | 4.0% (1.1–9.9%) | Self-Care |
| 3 | 64.0% (53.8–73.4%) | 47.0% (36.9–57.2%) | 54.0% (43.7–64.0%) | Non-Emergency |
| 4 | 34.0% (24.8–44.2%) | 60.0% (49.7–69.7%) | 50.0% (39.8–60.2%) | Self-Care |
| 5 | 78.0% (68.6–85.7%) | 78.0% (68.6–85.7%) | 82.0% (73.1–89.0%) | Non-Emergency |
| 6 | 22.0% (14.3–31.4%) | 51.0% (40.8–61.1%) | 41.0% (31.3–51.3%) | Self-Care |
| 7 | 67.0% (56.9–76.1%) | 61.0% (50.7–70.6%) | 64.0% (53.8–73.4%) | Non-Emergency |
| 8 | 100.0% (96.4–100.0%) | 100.0% (96.4–100.0%) | 100.0% (96.4–100.0%) | Non-Emergency |
| 9 | 100.0% (96.4–100.0%) | 98.0% (93.0–99.8%) | 96.0% (90.1–98.9%) | Emergency |
| 10 | 33.0% (23.9–43.1%) | 46.0% (36.0–56.3%) | 57.0% (46.7–66.9%) | Non-Emergency |
| 11 | 69.0% (59.0–77.9%) | 80.0% (70.8–87.3%) | 79.0% (69.7–86.5%) | Non-Emergency |
| 12 | 95.0% (88.7–98.4%) | 98.0% (93.0–99.8%) | 99.0% (94.6–100.0%) | Non-Emergency |
| 13 | 89.0% (81.2–94.4%) | 93.0% (86.1–97.1%) | 95.0% (88.7–98.4%) | Non-Emergency |
| 14 | 95.0% (88.7–98.4%) | 90.0% (82.4–95.1%) | 97.0% (91.5–99.4%) | Non-Emergency |
| 15 | 83.0% (74.2–89.8%) | 82.0% (73.1–89.0%) | 82.0% (73.1–89.0%) | Non-Emergency |
| 16 | 98.0% (93.0–99.8%) | 94.0% (87.4–97.8%) | 95.0% (88.7–98.4%) | Non-Emergency |
| 17 | 100.0% (96.4–100.0%) | 100.0% (96.4–100.0%) | 100.0% (96.4–100.0%) | Non-Emergency |
| 18 | 100.0% (96.4–100.0%) | 100.0% (96.4–100.0%) | 100.0% (96.4–100.0%) | Non-Emergency |
| 19 | 100.0% (96.4–100.0%) | 100.0% (96.4–100.0%) | 100.0% (96.4–100.0%) | Non-Emergency |
| 20 | 100.0% (96.4–100.0%) | 100.0% (96.4–100.0%) | 100.0% (96.4–100.0%) | Non-Emergency |
| 21 | 100.0% (96.4–100.0%) | 100.0% (96.4–100.0%) | 100.0% (96.4–100.0%) | Non-Emergency |
| 22 | 100.0% (96.4–100.0%) | 100.0% (96.4–100.0%) | 100.0% (96.4–100.0%) | Non-Emergency |
| 23 | 3.0% (0.6–8.5%) | 9.0% (4.2–16.4%) | 7.0% (2.9–13.9%) | Self-Care |
| 24 | 100.0% (96.4–100.0%) | 98.0% (93.0–99.8%) | 100.0% (96.4–100.0%) | Non-Emergency |
| 25 | 22.0% (14.3–31.4%) | 26.0% (17.7–35.7%) | 9.0% (4.2–16.4%) | Non-Emergency |
| 26 | 89.0% (81.2–94.4%) | 82.0% (73.1–89.0%) | 85.0% (76.5–91.4%) | Non-Emergency |
| 27 | 0.0% (0.0–3.6%) | 0.0% (0.0–3.6%) | 0.0% (0.0–3.6%) | Self-Care |
| 28 | 82.0% (73.1–89.0%) | 66.0% (55.8–75.2%) | 70.0% (60.0–78.8%) | Non-Emergency |
| 29 | 0.0% (0.0–3.6%) | 0.0% (0.0–3.6%) | 0.0% (0.0–3.6%) | Self-Care |
| 30 | 100.0% (96.4–100.0%) | 100.0% (96.4–100.0%) | 100.0% (96.4–100.0%) | Non-Emergency |
| 31 | 10.0% (4.9–17.6%) | 34.0% (24.8–44.2%) | 38.0% (28.5–48.3%) | Self-Care |
| 32 | 24.0% (16.0–33.6%) | 47.0% (36.9–57.2%) | 44.0% (34.1–54.3%) | Self-Care |
| 33 | 1.0% (0.0–5.4%) | 25.0% (16.9–34.7%) | 15.0% (8.6–23.5%) | Self-Care |
| 34 | 100.0% (96.4–100.0%) | 98.0% (93.0–99.8%) | 97.0% (91.5–99.4%) | Non-Emergency |
| 35 | 60.0% (49.7–69.7%) | 63.0% (52.8–72.4%) | 60.0% (49.7–69.7%) | Non-Emergency |
| 36 | 100.0% (96.4–100.0%) | 100.0% (96.4–100.0%) | 100.0% (96.4–100.0%) | Non-Emergency |
| 37 | 31.0% (22.1–41.0%) | 58.0% (47.7–67.8%) | 48.0% (37.9–58.2%) | Self-Care |
| 38 | 89.0% (81.2–94.4%) | 96.0% (90.1–98.9%) | 92.0% (84.8–96.5%) | Non-Emergency |
| 39 | 85.0% (76.5–91.4%) | 80.0% (70.8–87.3%) | 80.0% (70.8–87.3%) | Non-Emergency |
| 40 | 52.0% (41.8–62.1%) | 47.0% (36.9–57.2%) | 45.0% (35.0–55.3%) | Non-Emergency |
| 41 | 41.0% (31.3–51.3%) | 65.0% (54.8–74.3%) | 65.0% (54.8–74.3%) | Non-Emergency |
| 42 | 100.0% (96.4–100.0%) | 100.0% (96.4–100.0%) | 100.0% (96.4–100.0%) | Emergency |
| 43 | 83.0% (74.2–89.8%) | 69.0% (59.0–77.9%) | 77.0% (67.5–84.8%) | Non-Emergency |
| 44 | 8.0% (3.5–15.2%) | 32.0% (23.0–42.1%) | 21.0% (13.5–30.3%) | Self-Care |
| 45 | 0.0% (0.0–3.6%) | 0.0% (0.0–3.6%) | 0.0% (0.0–3.6%) | Self-Care |

Table S4. Sensitivity Analysis: Two models and their accuracy within each triage level across prompts with different temperature settings.

|  | Default Prompt, Default Temperature | Recognition-Primed Prompt, Default Temperature | Data/Frame-Prompt, Default Temperature | Default Prompt, Low Temperature | Recognition-Primed Prompt, Low Temperature | Data/Frame-Prompt, Low Temperature |
| --- | --- | --- | --- | --- | --- | --- |
| **Overall,**  **Mean (95% CI)** |  |  |  |  |  |  |
| Emergency (n=2) | 100.0% (91.2–100.0%) | 100.0% (91.2–100.0%) | 100.0% (91.2–100.0%) | 100.0% (91.2–100.0%) | 100.0% (91.2–100.0%) | 100.0% (91.2–100.0%) |
| Non-Emergency | 74.0% (70.3–77.3%) | 79.0% (75.6–82.1%) | 81.7% (78.4–84.6%) | 70.7% (66.9–74.2%) | 78.2% (74.7–81.3%) | 81.8% (78.5–84.7%) |
| Self-Care | 0.0% (0.0–1.5%) | 5.0% (2.9–8.4%) | 0.0% (0.0–1.5%) | 0.0% (0.0–1.5%) | 6.5% (4.1–10.2%) | 0.4% (0.1–2.1%) |
| **GPT 4.1 mini,**  **Mean (95% CI)** |  |  |  |  |  |  |
| Emergency (n=2) | 100.0% (83.9–100.0%) | 100.0% (83.9–100.0%) | 100.0% (83.9–100.0%) | 100.0% (83.9–100.0%) | 100.0% (83.9–100.0%) | 100.0% (83.9–100.0%) |
| Non-Emergency | 68.0% (62.5–73.0%) | 81.3% (76.5–85.3%) | 87.0% (82.7–90.3%) | 65.3% (59.8–70.5%) | 80.0% (75.1–84.1%) | 88.3% (84.2–91.5%) |
| Self-Care | 0.0% (0.0–2.9%) | 6.9% (3.7–12.6%) | 0.0% (0.0–2.9%) | 0.0% (0.0–2.9%) | 11.5% (7.1–18.2%) | 0.8% (0.1–4.2%) |
| **GPT 5.2 Instant,**  **Mean (95% CI)** |  |  |  |  |  |  |
| Emergency (n=2) | 100.0% (83.9–100.0%) | 100.0% (83.9–100.0%) | 100.0% (83.9–100.0%) | 100.0% (83.9–100.0%) | 100.0% (83.9–100.0%) | 100.0% (83.9–100.0%) |
| Non-Emergency | 80.0% (75.1–84.1%) | 76.7% (71.6–81.1%) | 76.3% (71.2–80.8%) | 76.0% (70.9–80.5%) | 76.3% (71.2–80.8%) | 75.3% (70.2–79.9%) |
| Self-Care | 0.0% (0.0–2.9%) | 3.1% (1.2–7.6%) | 0.0% (0.0–2.9%) | 0.0% (0.0–2.9%) | 1.5% (0.4–5.4%) | 0.0% (0.0–2.9%) |

Table S5. Sensitivity Analysis: Two models and their inter-rater reliability across prompts with different temperature settings.

|  | Default Prompt, Default Temperature | Recognition-Primed Prompt, Default Temperature | Data/Frame-Prompt, Default Temperature | Default Prompt, Low Temperature | Recognition-Primed Prompt, Low Temperature | Data/Frame-Prompt, Low Temperature |
| --- | --- | --- | --- | --- | --- | --- |
| GPT 4.1 mini | 0.824 | 0.634 | 0.756 | 0.873 | 0.651 | 0.761 |
| GPT 5.2 Instant | 0.926 | 0.742 | 0.775 | 0.920 | 0.812 | 0.815 |

Table S6. Sensitivity Analysis: Two models and the percentage of cases they solved correctly at least once among 10 trials across prompts with different temperature settings.

|  | Default Prompt, Default Temperature | Recognition-Primed Prompt, Default Temperature | Data/Frame-Prompt, Default Temperature | Default Prompt, Low Temperature | Recognition-Primed Prompt, Low Temperature | Data/Frame-Prompt, Low Temperature |
| --- | --- | --- | --- | --- | --- | --- |
| Overall, Mean (95% CI) | 57.8% (46.9-68.1%) | 74.4%  (64.2-83.1%) | 65.6% (54.8-75.3%) | 55.6% (44.7-66.0%) | 73.3%  (63.0-82.1%) | 65.6% (54.8-75.3%) |
| GPT 4.1 mini, Mean (95% CI) | 55.6% (40.0–70.4%) | 75.6% (60.5–87.1%) | 66.7% (51.0–80.0%) | 55.6% (40.0–70.4%) | 77.8% (62.9–88.8%) | 71.1% (55.7–83.6%) |
| GPT 5.2 Instant, Mean (95% CI) | 60.0% (44.3–74.3%) | 73.3% (58.1–85.4%) | 64.4% (48.8–78.1%) | 55.6% (40.0–70.4%) | 68.9% (53.4–81.8%) | 60.0% (44.3–74.3%) |
